# Endothelial Progenitor Cells Promote Directional Three-Dimensional Endothelial Network Formation by Secreting Vascular Endothelial Growth Factor

**DOI:** 10.1371/journal.pone.0082085

**Published:** 2013-12-03

**Authors:** Yoshinori Abe, Yoshiyuki Ozaki, Junichi Kasuya, Kimiko Yamamoto, Joji Ando, Ryo Sudo, Mariko Ikeda, Kazuo Tanishita

**Affiliations:** 1 School of Integrated Design Engineering, Graduate School of Science and Technology, Keio University, Hiyoshi, Kohoku, Yokohama, Japan; 2 School of Fundamental Science and Technology, Graduate School of Science and Technology, Keio University, Hiyoshi, Kohoku, Yokohama, Japan; 3 Department of Biological Engineering, Massachusetts Institute of Technology, Cambridge, Massachusetts, United States of America; 4 Laboratory of System Physiology, Department of Biomedical Engineering, Graduate School of Medicine, University of Tokyo, Tokyo, Japan; 5 Laboratory of Biomedical Engineering, School of Medicine, Dokkyo Medical University, Mibu, Tochigi, Japan; 6 Department of System Design Engineering, School of Science and Technology, Keio University, Hiyoshi, Kohoku, Yokohama, Japan; 7 Institute for Nanoscience and Nanotechnology, Waseda University, Wasedatsurumakicho, Shinjuku, Japan; University Heart Center Freiburg, Germany

## Abstract

Endothelial progenitor cell (EPC) transplantation induces the formation of new blood-vessel networks to supply nutrients and oxygen, and is feasible for the treatment of ischemia and cardiovascular diseases. However, the role of EPCs as a source of proangiogenic cytokines and consequent generators of an extracellular growth factor microenvironment in three-dimensional (3D) microvessel formation is not fully understood. We focused on the contribution of EPCs as a source of proangiogenic cytokines on 3D microvessel formation using an *in vitro* 3D network model. To create a 3D network model, EPCs isolated from rat bone marrow were sandwiched with double layers of collagen gel. Endothelial cells (ECs) were then cultured on top of the upper collagen gel layer. Quantitative analyses of EC network formation revealed that the length, number, and depth of the EC networks were significantly enhanced in a 3D model with ECs and EPCs compared to an EC monoculture. In addition, conditioned medium (CM) from the 3D model with ECs and EPCs promoted network formation compared to CM from an EC monoculture. We also confirmed that EPCs secreted vascular endothelial growth factor (VEGF). However, networks cultured with the CM were shallow and did not penetrate the collagen gel in great depth. Therefore, we conclude that EPCs contribute to 3D network formation at least through indirect incorporation by generating a local VEGF gradient. These results suggest that the location of EPCs is important for controlling directional 3D network formation in the field of tissue engineering.

## Introduction

Neovascularization is a crucial step in the development of reconstructed tissue applied to clinical practice. Although some two-dimensional (2D) tissue-engineered products such as skin and cornea substitutes have already been in practical use [1,2], reconstruction of three-dimensional (3D) tissues, such as the liver and heart, remains difficult. This is due to the need for 3D tissues to have an extensive vascular system to support tissues by providing oxygen and nutrients, as diffusion of the oxygen and nutrients cannot penetrate more than a few hundred microns. The difficulty of providing a blood supply has limited the size of 3D engineered tissues *in vitro*. Therefore, great demand exists for the vascularization of engineered tissues *in vitro*.

To achieve the fairly uniform distribution of microvessels inside tissue-engineered organoids, newly formed microvessels should penetrate deep inside the organoids. Recent findings recognize that the concentration profile of extracellular growth factors, such as vascular endothelial growth factor (VEGF) and basic fibroblast growth factor (bFGF), plays an important role in regulating the formation of 3D microvascular networks. For example, a steep concentration gradient of VEGF secreted from neural tubes attracted the tips of endothelial cell (EC) filopodia, thereby controlling the vascular branching pattern during embryogenesis [3]. In addition, Gerhardt et al. [4] showed that endothelial tip cells detected the VEGF gradient generated by retinal astrocytes and migrated along the VEGF distribution, resulting in a continuous directional vascular formation. These findings *in vivo* indicate that 3D microvessel formation *in vitro* can also be controlled by the concentration profile of extracellular growth factors generated by cells. Therefore, an appropriate cell source for continuous secretion of extracellular growth factors is needed to regulate 3D microvessel formation *in vitro*.

Recently, the contributions of endothelial progenitor cells (EPCs) have attracted attention in inducing vascularization for tissue engineering applications, and especially for clinical application with autologous cell transplantation [5]. EPCs are easily isolated from peripheral blood, bone barrow, and umbilical cord blood [6–8] as mononuclear hematopoietic progenitor cells that have differentiated into ECs in culture [9]. Previous studies have demonstrated that EPCs accumulate in active angiogenic region [10–12] and contribute directly for the treatment of critical ischemia through participation in neovascularization [13–15]. However, most EPCs that accumulate in angiogenic regions do not always cooperate directly for the formation of the vasculature, but rather reside in the tissue and have the potential to contribute indirectly [16,17]. In addition, previous studies have demonstrated that cultured EPCs secrete various proangiogenic cytokines such as VEGF, endothelial nitric oxide synthase (eNOS), inducible nitric oxide synthase (iNOS) [16], insulin-like growth factor 1 (IGF-1), stromal-derived factor 1 (SDF-1) [17], hepatocyte growth factor (HGF), and granulocyte colony-stimulating factor (G-CSF) [18]. The indirect contribution of EPCs to neovessel formation indicates that the cells can act as a source of proangiogenic cytokines to induce vascularization of tissue-engineered organoids *in vitro*. However, most recent studies have been performed in conventional 2D culture models [19,20]. Although 3D extracellular matrices (ECMs) store various proangiogenic cytokines and generate a concentration gradient of VEGF for directional 3D microvessel formation in the microenvironment [21], the indirect role of EPCs as a source of proangiogenic cytokines in a 3D environment with ECMs remains unclear. Koga et al. [22] revealed that EPC contributed to 3D network formation predominantly by creating local growth factor concentration with direct incorporation. Therefore, using a 3D model that mimics the *in vivo* microenvironment is necessary to investigate the indirect contribution of EPCs as a source of proangiogenic cytokines. 

In this study, we clarified the indirect contribution of EPCs as a source of proangiogenic cytokines on 3D microvessel formation using an *in vitro* 3D model. A 3D coculture model with ECs and EPCs promoted extensive EC network formation and allowed networks to invade deeper into the gel compared to an EC monoculture. In addition, we focused on the dependency of the concentration of bFGF on 3D endothelial network formation. A coculture model with a high concentration of bFGF synergistically increased the length and number of networks in the horizontal direction compared with that using a low concentration of bFGF and an EC monoculture. Although EPC conditioned medium (CM), in which EPCs secreted VEGF, also promoted network formation in the horizontal direction, the CM did not promote an invasion of EC networks deep into the collagen gel. Therefore, this study demonstrated that EPCs secrete VEGF and induce network invasion into gels three-dimensionally by creating a local VEGF gradient.

## Materials and Methods

### Ethics Statement

All animals used in the experiments received humane care and the experimental protocol was approved by the Committee of Laboratory Animals following Keio University guidelines.

### EPC Isolation and Culture

EPCs were isolated from rat bone marrow as described previously [22]. Bone marrow was obtained by flushing tibiae and femurs of Sprague–Dawley rats (250–300 g; Nippon Bio-Supply Center, Tokyo, Japan). Mononuclear cells were isolated from bone marrow by density gradient centrifugation using Histopaque-1083 (Sigma-Aldrich, St. Louis, MO, USA). The mononuclear cells were seeded onto 35-mm cell culture dishes (Corning, Corning, NY, USA) pre-coated with vitronectin (2.5 µg/ml, Sigma-Aldrich) at 2.5 × 10^6^ cells/cm^2^ and cultured with EGM-2MV (Lonza, Walkersville, MD, USA) supplemented with 20% fetal bovine serum (FBS) and EGM-2MV SingleQuots (Lonza) without hydrocortisone and FBS under standard conditions (37°C, 5% CO_2_). After 5 days of culture, nonadherent cells were discarded by double rinses with phosphate-buffered saline (PBS) and the culture medium was replaced. After 7 days, the cultured cells were confirmed to be EPCs by detecting the presence of both low-density lipoprotein acetylated DiI complex (Invitrogen, Carlsbad, CA, USA) and FITC-labeled Ulex europaeus agglutinin I (Sigma-Aldrich), and forming 2D networks on Matrigel (BD Biosciences, Bedford, MA, USA), which have been commonly referred to as EPC characteristics, as demonstrated previously [22]. 

### EC Culture

Bovine pulmonary microvascular endothelial cells (BPMECs) were purchased from Cell Systems Corp. (Kirkland, WA, USA). BPMECs were cultured in Dulbecco’s modified Eagle’s medium (DMEM; Invitrogen) containing 10% FBS, 1% antibiotic–antimycotic (Invitrogen), and 15 mM HEPES (Sigma-Aldrich), and used at passages 9–10 in our experiments.

### *In Vitro* 3D Network Models

**Figure 1:**
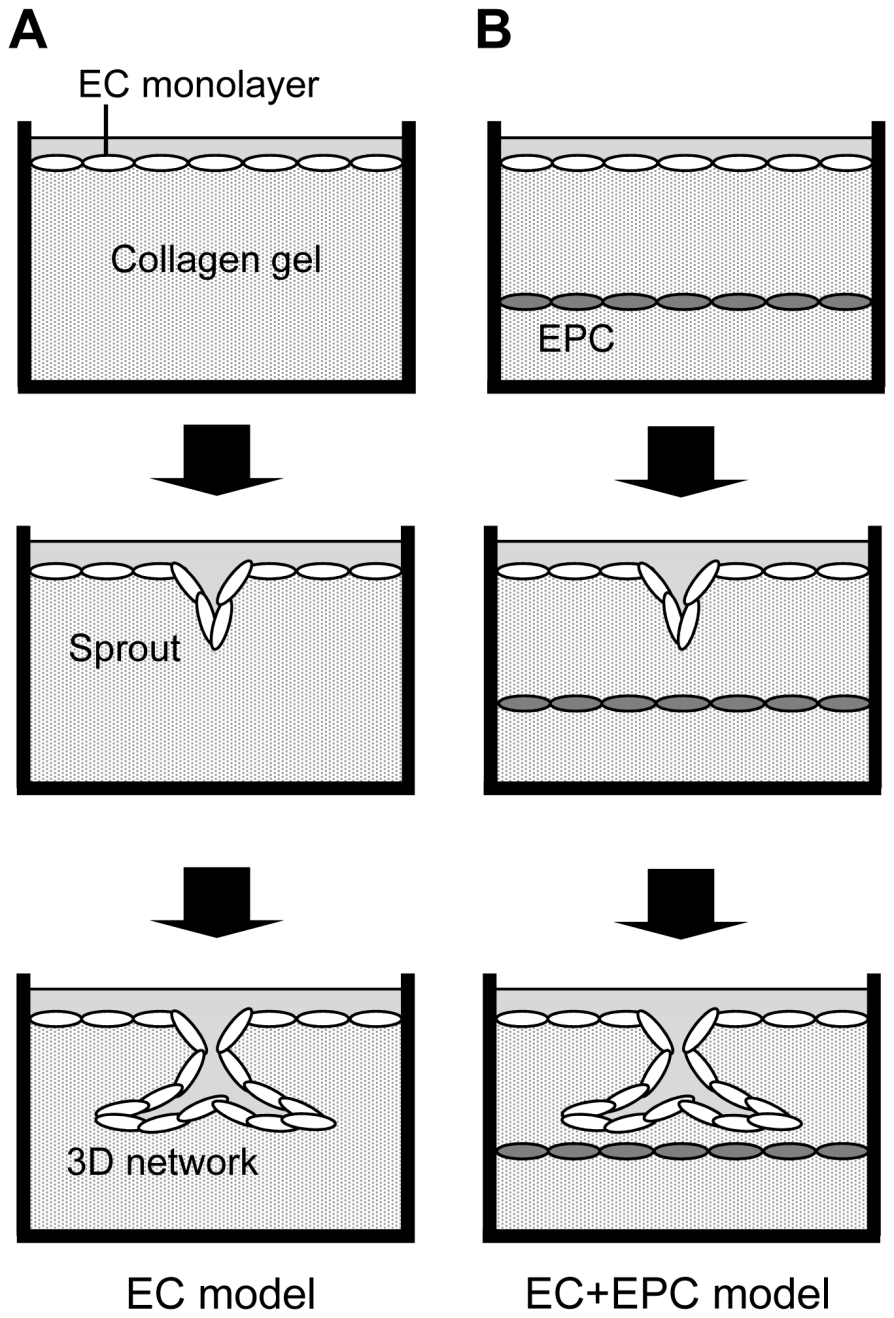
Three-dimensional endothelial network models. (**A**) In the EC model, ECs were seeded onto collagen gel. The EC monoculture served as a control. (**B**) In the EC+EPC model, EPCs were sandwiched with double layers of collagen gel. ECs were then cultured on the top of the upper collagen gel layer. In each model, some ECs in a confluent monolayer invaded the underlying collagen gel with the addition of bFGF (Sprout) and formed 3D endothelial networks in culture (3D network).

3D network models were prepared to form microvessel-like networks (Figure 1A) as described previously [22]. The collagen was prepared as follows: 3 mg/mL type I collagen solution (Nitta Gelatin, Osaka, Japan) was mixed with 10 × minimum essential medium (Invitrogen) and 0.08 N NaOH (8:1:1; Wako Pure Chemical, Tokyo, Japan) on ice. The mixture was poured into a glass-base dish (Asahi Glass, Tokyo, Japan) and polymerized at 37°C for 1 h. BPMECs were seeded onto collagen gel dishes at 3 × 10^4^ cells/cm^2^. After 24 h, the medium was changed. The cells reached 80% sub-confluence 48 h after seeding. DMEM was then changed to EGM-2MV+DMEM, a mixture of EGM-2MV and DMEM in a 1:1 ratio. EGM-2MV+DMEM was supplemented with 10 ng/mL bFGF (PeproTech, Rocky Hill, NJ, USA) to induce network formation. “Day 0” was defined as the first day of confirmed initial sprouting of EC networks. This model was referred to as the “EC model.” The medium was changed every other day.

To reveal the ability of proangiogenic factors secreted by EPCs on the 3D network model, EPCs were embedded in collagen gel using the EC model (Figure 1B). EPCs cultured for 7 days were isolated from dishes using trypsin–EDTA (Invitrogen) and seeded at 2 × 10^4^ cells/cm^2^ in 500 μL collagen gel. Nonadherent cells were discarded by double rinses with PBS 24 h after seeding. Next, the cells were sandwiched with 200 μL collagen gel. BPMECs were seeded onto collagen gel 1 h after incubation. After 24 h, the medium was changed. The DMEM was changed to EGM-2MV+DMEM 48 h after seeding. This model was referred to as the “EC+EPC model.” The medium was changed every other day. The thickness of the collagen gel between the EC and EPC layers was 277 ± 41 μm.

### Analysis of the *In Vitro* 3D Network

ECs, stimulated with 10 ng/mL bFGF in the 3D model, invaded into the collagen gel layer and formed microvessel-like network structures. The 3-D networks were photographed by using a bright-field/phase-contrast microscope for 5 days. The images were randomly selected, and the outline images of vessel network was traced manually from selected images and binarized. Then the images were skeletonized by eliminating the pixel to form the center line of individual vessel. These procedures were achieved by software of Image J (National Institutes of Health, Bethesda, MD, USA).

We measured the total of the length of all skeletonized networks in field, and counted the number of all networks in field. For example, the network, formed by connection from two networks, was counted as one network. The morphology of vessels and cells disappeared in the skeletonized procedure of binarized images. In this study we focused on the growth of 3D network based on the skeletonized images.

### Analysis of bFGF Contribution on Network Formation

Using EGM-2MV+DMEM supplemented with 10 and 30 ng/mL bFGF, the effect of bFGF concentration on network formation was investigated. After 5 days in culture, the network structures were photographed and measured for the length and number of networks in each image by the same procedure mentioned above.

### Depth Analysis of the 3D Network

To analyze the depth of 3D network structures, cells in 3D network models were dyed with 25 mM CellTracker Green BODIPY (Invitrogen) and incubated for 45 min at 37°C after 5 days in culture. EC and EPC were then fixed with 3.7% paraformaldehyde in PBS for 15 min at room temperature. Images were obtained at 2.5-µm-depth intervals from the confluent EC layer on the gel to an apical network formation or EPC layer in the gel using a confocal laser scanning microscope (Carl Zeiss, Hallbergmoos, Germany). Three to five networks were randomly selected per culture dish, and the depths of networks were quantitatively analyzed. LSM Image Browser (Carl Zeiss) and Imaris (Bitplane AG, Zurich, Switzerland) were used for image data processing.

### Effect of CM on Network Formation

To confirm the effect of growth factors secreted by EPCs on 3D network formation, CM from the EC and EC+EPC models were collected at days 2 and 4 after culture. These CMs were mixed with fresh EGM-2MV+DMEM supplemented with 30 ng/mL bFGF (1:1) and applied to the EC model after which BPMECs had reached confluency. After 5 days in culture, the 3D networks were photographed and analyzed for the length of networks in each image. The medium was changed every day.

### Analysis of VEGF Secretion by EPCs

To analyze whether EPCs secrete VEGF, the 3D networks models were cultured in EGM-2MV+DMEM supplemented with 30 ng/mL bFGF. After 4 or 5 days in culture, CM from the EC and EC+EPC models was collected. Because EPCs were embedded in the collagen gel, VEGF concentration in the collagen gel may have a higher level than that in CM. Therefore, VEGF concentration in the collagen gel was also assayed under the EC+EPC model. After 4 or 5 days in culture, collagen gel in the EC+EPC model was lysed by 3mg/mL collagenase solution (Sigma-Aldrich), incubated for 30 minutes at 37°C and collected. The concentration of VEGF was assayed using a rat enzyme-linked immunosorbent assay (ELISA) kit (RayBiotech, Norcross, GA, USA). 

### Statistical Analysis

Data are presented as the means ± standard deviation (SD) for each group. At least three independent experiments were performed for all analyses. A Student’s t-test was used to test for differences between the two groups. In addition, the significance of differences among three or more groups was compared using Scheffe’s method. A value of *p* < 0.05 was considered to indicate statistical significance.

## Results

### *In Vitro* 3D Endothelial Network Formation

**Figure 2:**
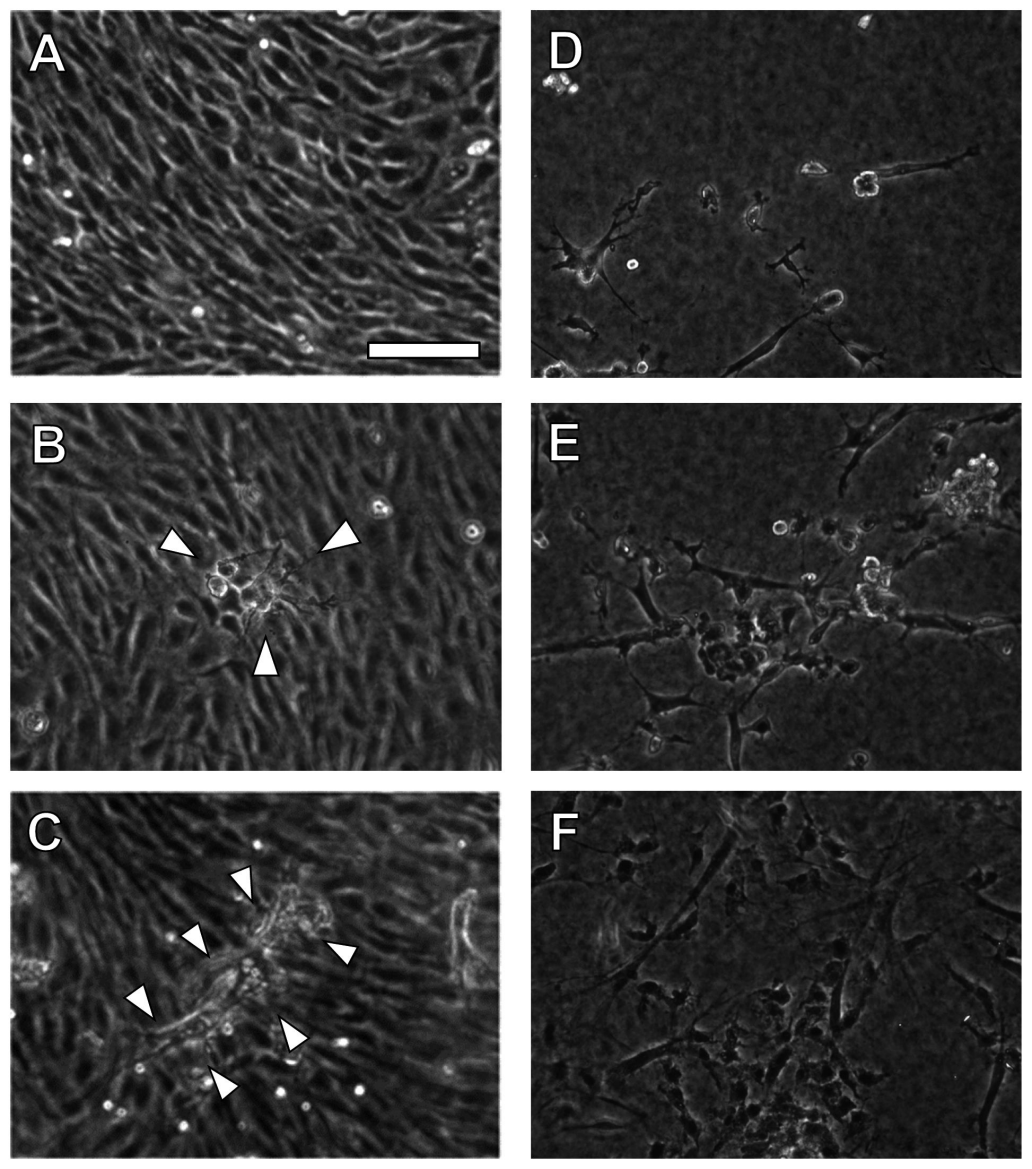
EC network formation and growth of EPCs in EC and EC+EPC models. In the presence of bFGF, ECs formed a confluent monolayer on the gel (**A**) and invaded into the underlying gel (**B**). After 5 days in culture, ECs formed longer EC networks (**C**, arrowheads). EPCs embedded between double collagen gel layers gradually proliferated in culture (**D**: day 1, E: day 3, F: day 5). Scale bar, 100 μm.

To clarify the effect of EPCs on 3D endothelial network formation, an *in vitro* 3D network model was used. After bFGF was added to the sub-confluent EC layer, the growing process of 3D endothelial networks was examined using phase-contrast images. The ECs formed a confluent monolayer on the collagen gel (Figure 2A) and began to invade underlying collagen gel (Figure 2B, arrowheads). ECs formed microvessel-like networks in the collagen gel in the presence of bFGF (Figure 2C, arrowheads). In the EC+EPC model, EPCs embedded between double collagen gel layers and gradually proliferated in culture (Figure 2D–F).

**Figure 3:**
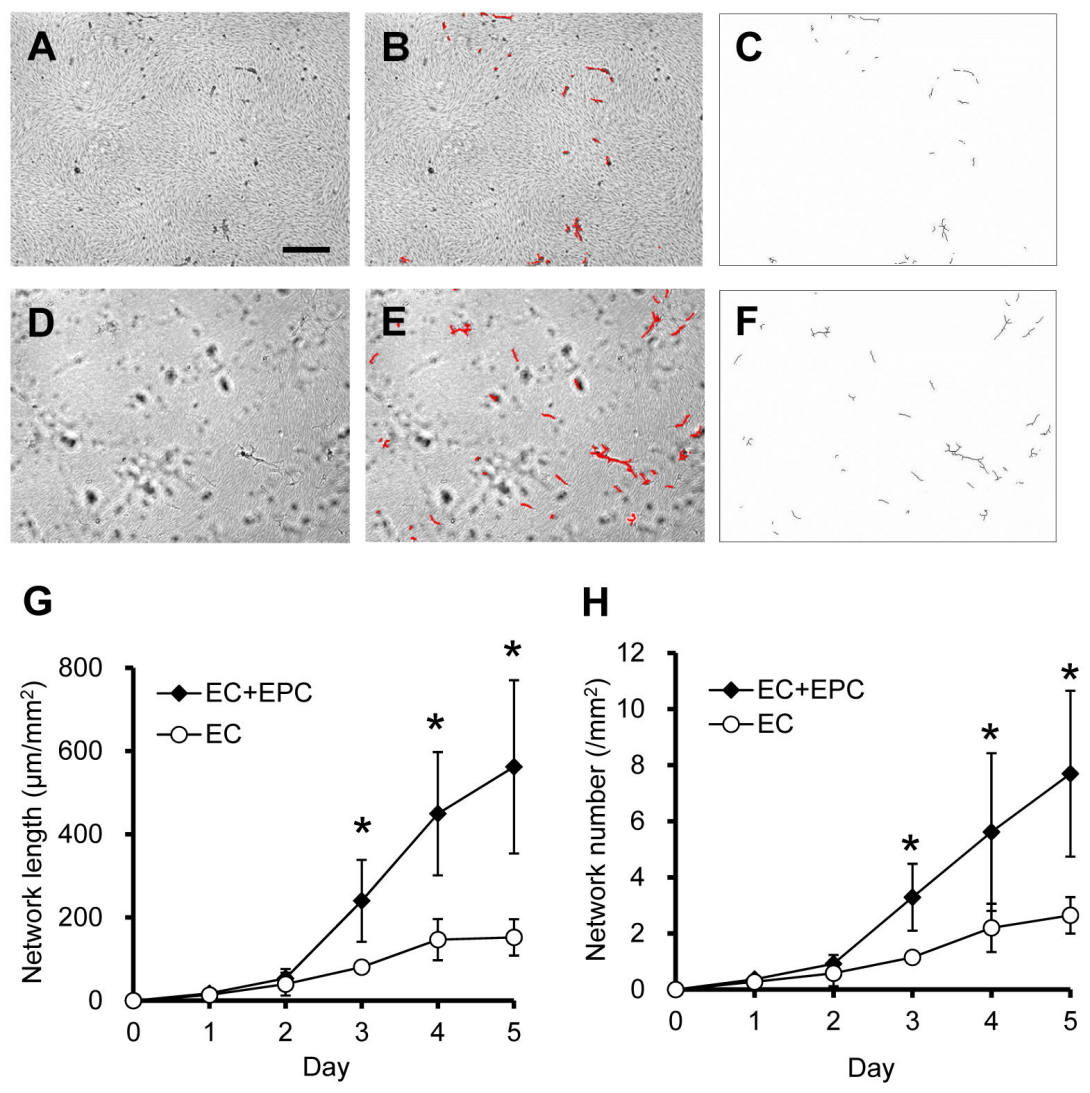
EC network formation in the EC and EC+EPC models. After 5 days in culture, bright-field images indicate EC network formation in the EC (**A**) and EC+EPC models (D) with 10 ng/mL bFGF. Using these images, EC networks were marked in red (**B**, **E**) and skeletonized (**C**, **F**), and the length and number of the EC networks were measured. The EC+EPC model significantly increased both the length (**G**) and number (**H**) of EC networks compared to the EC model at days 3–5. Data are presented as the means ± SD (n = 27–51; *p < 0.05 vs. EC model). Scale bar, 300 μm.

The growth of networks in 3D models was monitored using bright-field microscopy for 5 days. After 5 days in culture, bright-field images indicated network formation in the EC (Figure 3A) and EC+EPC models (Figure 3D) with 10 ng/mL bFGF. Using these images, EC networks were marked in red (Figure 3B, E) and skeletonized (Figure 3C, F), and the length and number of networks were quantitatively evaluated. The length of networks in the EC+EPC model was about three times longer than that in the EC model at days 3–5 (Figure 3G). The number of networks in the EC+EPC model reached 8 ± 3 networks/mm^2^, whereas that of the EC model was 3 ± 1 networks/mm^2^ at day 5 (Figure 3H). These results indicate that the ECs in the EC+EPC model frequently invade the underlying collagen gel to construct 3D endothelial networks. 

### Effect of bFGF Contribution to EC Network Formation

**Figure 4:**
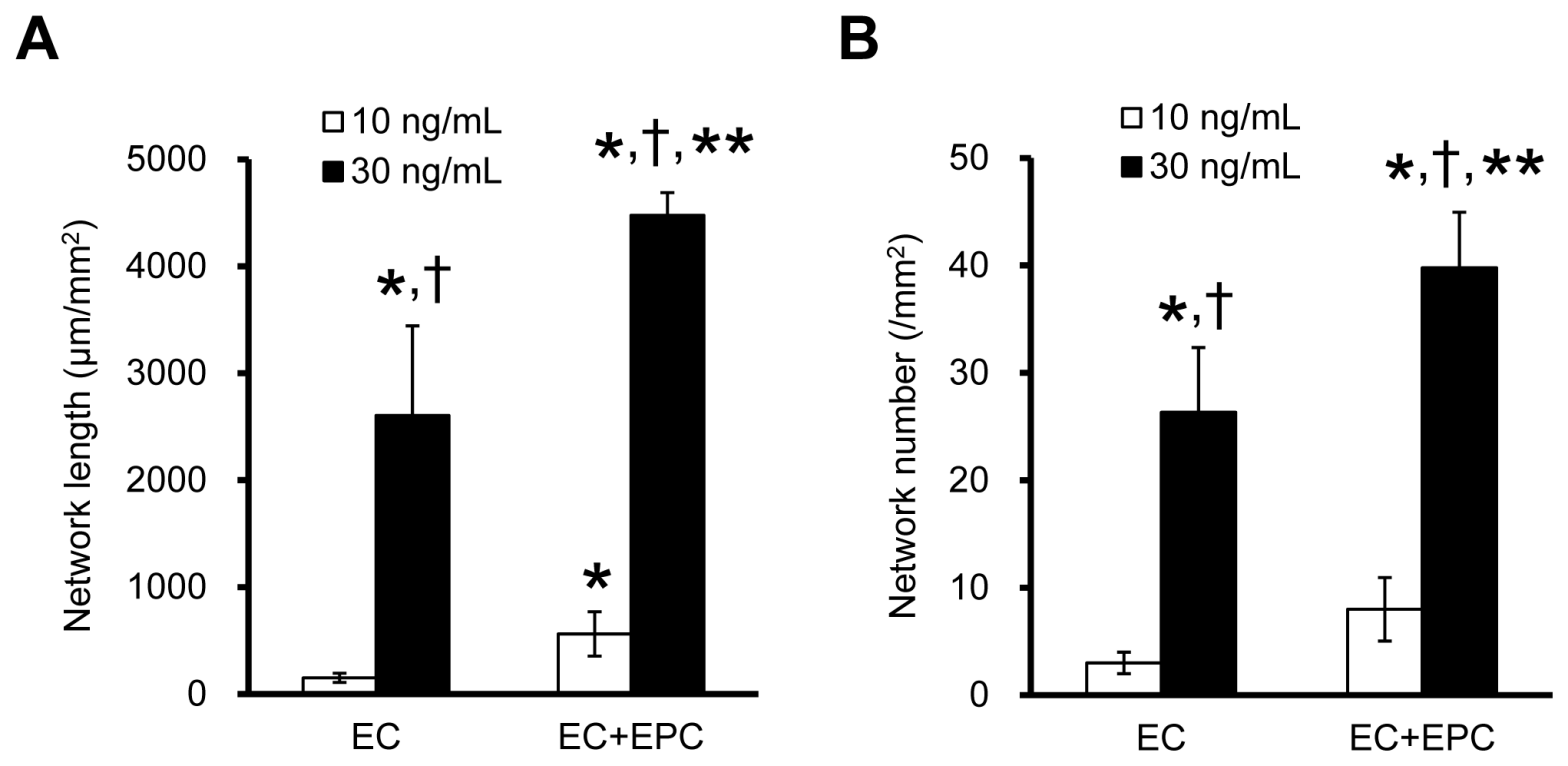
Effect of supplemented bFGF on EC network formation. The EC and EC+EPC models with 30 ng/mL bFGF significantly increased both the length (**A**) and number (**B**) of EC networks compared to 10 ng/mL bFGF at day 5. Furthermore, the EC+EPC model with 30 ng/mL bFGF enhanced the length (**A**) and number (**B**) of EC networks to a greater extent than the EC model with 30 ng/mL bFGF. Data are presented as the means ± SD (n = 8–51; *p < 0.03 vs. EC model with 10 ng/mL bFGF; ^†^ *p* < 0.001 vs. EC+EPC model with 10 ng/mL bFGF; **p < 0.001 vs. EC model with 30 ng/mL bFGF).

The EC and EC+EPC models were also cultured in EGM-2MV+DMEM supplemented with 30 ng/mL bFGF. The length and number of network structures in the EC and EC+EPC models with 10 and 30 ng/mL bFGF were compared (Figure 4). The EC model with 30 ng/mL bFGF significantly increased the length and number of network structures compared to the EC and EC+EPC models with 10 ng/mL bFGF (Figure 4). Moreover, the EC+EPC model with 30 ng/mL bFGF synergistically enhanced the length and number of networks at a greater extent than the EC model with 30 ng/mL bFGF (Figure 4). These results show that a high concentration of bFGF enhances EC network formation in the horizontal direction in each model, as well as the synergistic effects of EPCs and bFGF on 3D network formation.

### Depth Distribution of 3D Endothelial Networks

**Figure 5:**
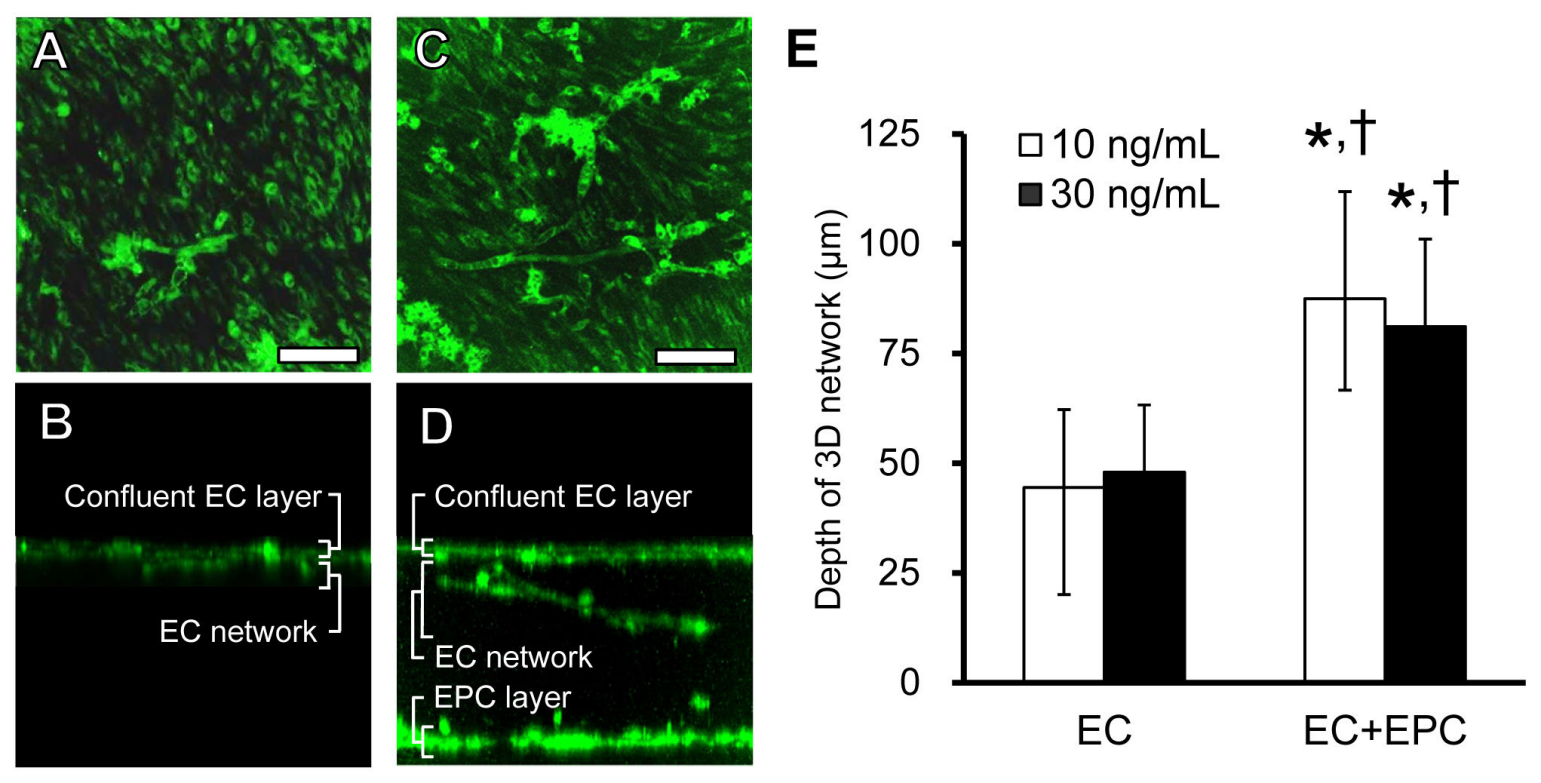
3D endothelial network formation in the depth direction in EC and EC+EPC models. Representative fluorescent images of a confluent EC layer on the surface of collagen gel (**A**, **C**) and EC networks in the collagen gel (**B**, **D**) are shown. (**E**) The depth of the EC and EC+EPC models was quantitatively analyzed. The depth of 3D endothelial networks in the EC+EPC model was deeper than that in the EC model. However, the difference was not remarkable according to the concentration of bFGF. Data are presented as the means ± SD (n = 6–26; *p < 0.001 vs. EC model with 10 ng/mL bFGF; ^†^ *p* < 0.001 vs. EC model with 30 ng/mL bFGF). Scale bar, 100 μm.

To observe the network structure of 3D models in detail, ECs were dyed with green fluorescent CellTracker after 5 days in culture and three-dimensionally analyzed using a confocal laser-scanning microscope. Representative fluorescent images were three-dimensionally reconstructed by calculating 2.5-µm-depth-interval images. Z-axis projection images show a confluent EC layer on the surface of the collagen gel (Figure 5A, C). Cross-sectional images of 3D models show EC networks (Figure 5B, D) and an EPC layer (Figure 5D). These images indicate that network structures in 3D models expand into the underlying collagen gel from the confluent EC layer. The EC+EPC model was successful in forming many stable networks in greater depth (Figure 5D). 

Next, the effect of EPCs on 3D network formation was characterized by quantifying the invading network depth. In the presence of 10 ng/mL bFGF, the depths of 3D networks in the EC and EC+EPC models were 44.4 ± 17.8 μm and 87.5 ± 24.4 μm, respectively (Figure 5E). Moreover, in the presence of 30 ng/mL bFGF, the depths of 3D networks in the EC and EC+EPC models were 48.0 ± 15.3 μm and 81.1 ± 19.9 μm, respectively (Figure 5E). The networks in the EC+EPC model invaded about twofold deeper than those of the EC model (Figure 5E). However, no significant difference was observed between the depths of networks with regard to bFGF concentration (Figure 5E).

### Effect of CM on Network Formation

**Figure 6:**
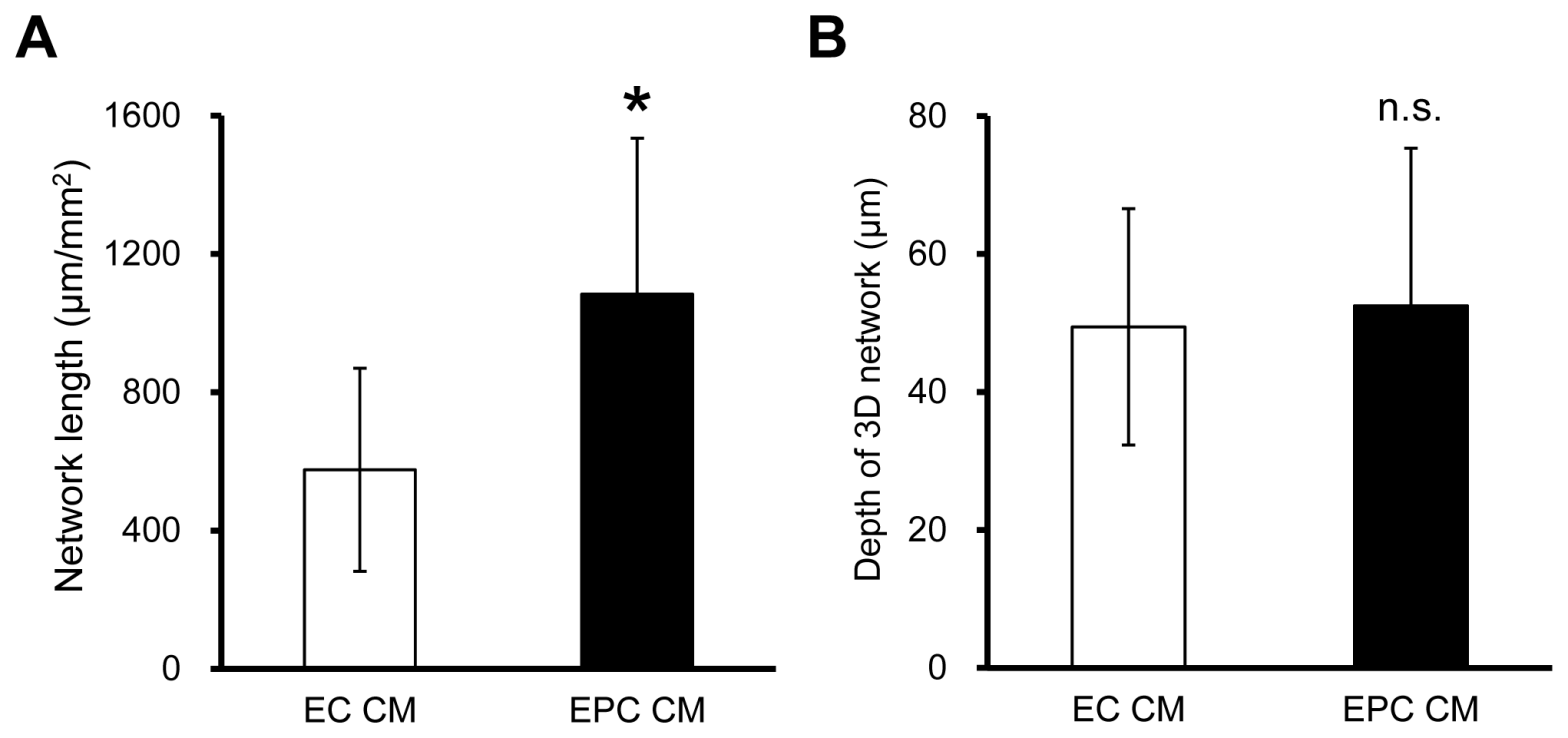
Effect of soluble factors secreted from EPCs on EC network formation in the EC model. CM from the EC+EPC model developed networks earlier than that of the EC+EPC model. After 5 days in culture, the length of networks in CM from the EC+EPC model was about twice longer than that from the EC model (**A**; n = 6). However, the difference was not significant in the depth of 3D endothelial networks (**B**; n = 8). Data are presented as the means ± SD (*p < 0.05 vs. CM from the EC model).

The continuous supply of growth factors from EPCs promotes EC network formation. To explore the effect of growth factors in the EC+EPC model on network formation, the EC model was cultured with CM from both the EC and EC+EPC models at days 2 and 4 after culture. After 5 days in culture, quantitative analysis showed that CM from the EC+EPC model induced EC networks about twice longer than those of the EC model (Figure 6A). The depth of EC networks in these models was also quantitatively analyzed. However, no significant difference was observed between the depths of EC networks with regard to CM (Figure 6B).

### VEGF Secretion in 3D Network Models

**Figure 7:**
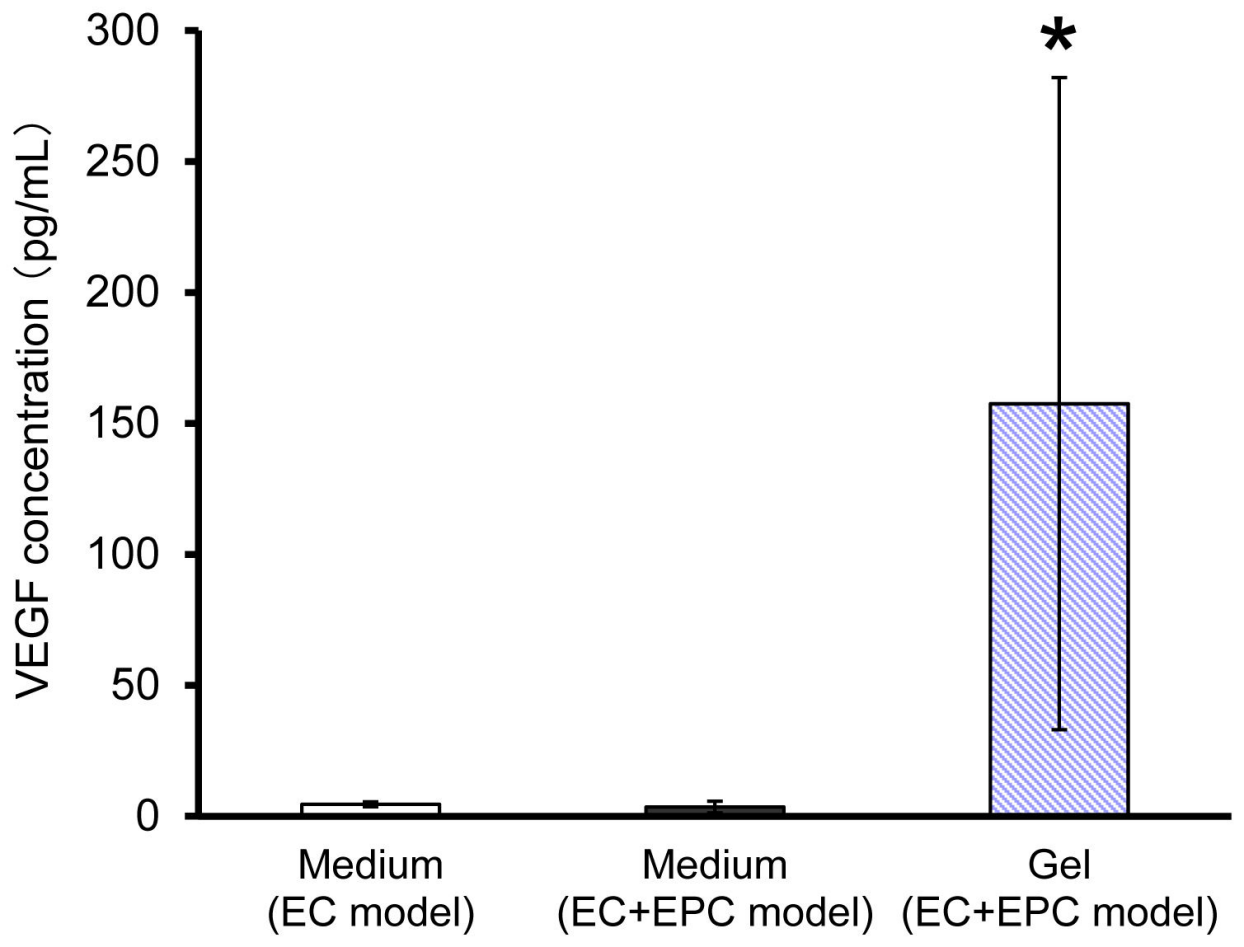
VEGF concentration in 3D endothelial network models. CM from the EC and EC+EPC models and collagen gel from the EC+EPC model were collected and analyzed by ELISA to compare VEGF concentrations. The concentration of VEGF in collagen gel was significantly more than that in CM from the EC and EC+EPC models. Data are presented as the means ± SD (n = 3–8; *p < 0.03 versus CM from EC and EC+EPC models).

VEGF enhances directional branching, invasion, and migration of endothelial tip cells from existing vessels [4,23]. Here, we focused on VEGF secretion by EPCs as the mechanism that contributes to angiogenic effects. Results from ELISA showed that VEGF concentration in the collagen gel was 157.6 pg/mL in the EC+EPC model and was detected significantly more than CM from the EC and EC+EPC models (Figure 7). In contrast, VEGF concentration between CM from the EC and EC+EPC models did not cause a significant difference (Figure 7).

## Discussion

### *In Vitro* 3D Model to Investigate Directional Network Formation

To clarify the indirect effects of EPCs on angiogenic endothelial sprouting, various EC-EPC coculture models have been developed. Wang et al. [20] developed 2D coculture models to elucidate whether EPCs control the vascular morphogenesis as a source of proangiogenic cytokines. In addition, Scheubel et al. [24] designed an *in vitro* 3D model in which EPCs and EC spheroids embedded in collagen gel were separately cultured but shared a common medium to generate a paracrine diffusion gradient within the media. Although EPCs attract a directional 3D network formation through a common medium, EPCs residing in the tissue provide ECs with growth factors that are distributed in the ECM and promote EC migration and proliferation in physiological events [5]. Growth factor results in different behavior between collagen gel and medium, and VEGF immobilized in a collagen scaffold promotes penetration and proliferation of ECs compared to medium supplemented with VEGF [25]. However, little is known about the effect of proangiogenic cytokines secreted from EPCs in collagen gel on 3D endothelial network formation. In this study, indirect contribution of EPCs as a source of proangiogenic cytokines on 3D microvessel formation was investigated using an *in vitro* 3D coculture model. This study is the first to demonstrate that EPCs embedded in collagen gel promote 3D endothelial network formation through the secretion of proangiogenic cytokines such as VEGF.

### Effect of EPCs on 3D Network Growth

In the EC+EPC model, the length and number of EC networks increased significantly at days 3–5 compared to the EC model when 10 ng/mL bFGF was supplemented. This increase in the length and number of EC networks with EPCs was also observed at a higher concentration of bFGF (30 ng/mL), suggesting that EPCs secrete growth factors other than bFGF to enhance EC network formation. However, a high concentration of bFGF (30 ng/mL) in the EC+EPC model significantly increased the length and number of EC networks compared to a low concentration of bFGF (10 ng/mL) in the EC and EC+EPC models and a high concentration of bFGF in the EC model. 

Angiogenic cytokines such as bFGF [26–28] and VEGF [28,29] enhance angiogenesis. Jeon et al. [30] demonstrated that the combined therapy of sustained delivery of bFGF and EPC mobilization with granulocyte colony-stimulating factor (G-CSF) administration potentiated the angiogenic efficacy in an *in vivo* mouse hindlimb ischemia model. Furthermore, our previous study showed that EPCs isolated from rat bone marrow contribute to *in vitro* 3D endothelial network formation using medium supplemented with 20 ng/mL bFGF [22], although little is known about the effect of concentration of angiogenic cytokines on EC network formation with EPCs. The present experiments demonstrated that the combination of coculture with EPCs and a high concentration of bFGF can synergistically enhance network formation and is extremely useful for the vascularization of engineered tissues *in vitro*. 

### Effect of the 3D Concentration Gradient of VEGF Secreted by EPCs

Paracrine attraction of vascular growth requires the release of proangiogenic cytokines, such as VEGF [5]. A concentration gradient of VEGF activates ECs of existing blood vessels and guides directional sprout growth [4,31]. However, while a previous study indicated the paracrine effect of EPCs on angiogenic endothelial sprouting using an *in vitro* 3D model, proangiogenic cytokines such as VEGF produced by EPCs was below the sensitivity range of the multiplex cytometric bead array because the model was not designed to evaluate which specific factors of EPCs induced sprout formation [24]. 

Here, we focused on VEGF, secreted by EPCs, as a candidate contributing to angiogenic effects. An ELISA for VEGF revealed that VEGF concentration in the collagen gel embedding EPCs was detected significantly compared to CM from the EC and EC+EPC models. An ELISA kit used in this experiment is effective to the rat EPC, and some uncertainty may exist in the measurement of VEGF for bovine EC. However, the previous study of our group [22] revealed that VEGF secreted from EPC was significant by VEGF neutralization in the EPC+EC model. The bioactivity of the secreted VEGF was neutralized by recombinant VEGF receptor 1. The VEGF neutralization led to attenuation in the network formation in the EPC+EC model. The network formation in the EC model was lower than that of EPC+EC model with neutralized VEGF [22], indicating that VEGF secreted from EC was very slight. Thus VEGF secreted from EPC could be prominent compared to that from EC. 

Furthermore, to test the effect of soluble factors secreted by EPCs on 3D endothelial network formation, the effect of CM from the EC+EPC model on 3D endothelial network formation was analyzed. CM from the EC+EPC model promoted the length of EC networks compared to that from the EC model. In contrast, CM from the EC+EPC model induced no significant difference in the depth of 3D endothelial networks. These results suggest that distribution, rather than concentration, of angiogenic growth factors is important to induce deeper EC networks. Therefore, our findings demonstrate that the EC+EPC model can induce 3D endothelial network formation toward a locally higher concentration of proangiogenic cytokines, such as VEGF secreted by EPCs. Santo et al. [32] reported that intramuscular injection of EPC-derived CM is as effective as cell transplantation for promoting tissue revascularization and functional recovery. However, our results suggest that the location of EPCs and consequent local concentration gradient of growth factors secreted by EPCs are important in controlling directional 3D endothelial network formation.

## Conclusions

This study demonstrated the contribution of EPCs as a source of proangiogenic cytokines on 3D endothelial network formation using an *in vitro* 3D network model. The EC+EPC model significantly promoted network formation not only in the horizontal direction but also deeper into the collagen gel compared to the EC monoculture model. In addition, CM from the EC+EPC model promoted endothelial network formation compared to that from the EC model, and VEGF was significantly detected in CM from EPCs rather than fresh medium. Therefore, EPCs secrete proangiogenic factors such as VEGF and control directional 3D endothelial network formation. The present culture model is useful for understanding the mechanism of EPCs as a source of proangiogenic cytokines on 3D microvessel formation.
